# LCMS dataset on compounds in *Syzygium polyanthum* (Wight) Walp. leaves variant from the East coast of Peninsular Malaysia

**DOI:** 10.1016/j.dib.2021.107485

**Published:** 2021-10-17

**Authors:** T.A. Faiz T. Anuar, Azlini Ismail, Izzat Fahimuddin Mohamed Suffian, Azzmer Azzar Abdul Hamid, Mohd Hafiz Arzmi, Muhammad Nor Omar

**Affiliations:** a Department of Chemistry, Kulliyyah of Science, International Islamic University Malaysia, Kuantan, Pahang 25200, Malaysia; b Department of Fundamental Dental and Medical Sciences, Kulliyah of Dentistry, International Islamic University Malaysia, Kuantan, Pahang 25200, Malaysia; c Department of Pharmaceutical Chemistry, Kulliyyah of Pharmacy, International Islamic University Malaysia, Kuantan, Pahang 25200, Malaysia; d Department of Biotechnology, Kulliyyah of Science, International Islamic University Malaysia, Kuantan, Pahang 25200, Malaysia

**Keywords:** *Syzygium polyanthum*, LCMS, Serai kayu, Salam, Traditional plant

## Abstract

The data presented here is the liquid chromatography and mass spectrometry (LC-MS) profile of phytochemical compounds in the aqueous extract of *Syzygium polyanthum* (Wight) Walp. leaves. This plant is consumed raw and sometimes added to local dishes of people in Southeast Asia countries. Most importantly, it has ethnomedicinal values mainly in treating diabetes and hypertension, and at the same time, this plant has anti-microbial, anti-oxidant, anti-cancer, and anti-tumor properties [1]. There are chemical composition variations reported between the same species of different geographical locations, which eventually affect the plant's therapeutic potential [2], [3]. This dataset represents the identified compounds for *S. polyanthum* (Wight) Walp. leaves, a variant collected from Kuantan, a city located in the Pahang state on the East Coast of Peninsular Malaysia. The leaves were then dried in an open-air at room temperature for three weeks, ground, and then macerated in water inside a bath-sonicator, freeze-dried, and then run using LCMS. The LCMS was run using the ultra-performance liquid chromatography equipped with an electrospray time-of-flight mass spectrometer detector, operated in a negative-ion mode. The mass spectral features from samples raw data were matched with Traditional Medicine (en) and Waters Screening libraries in the Waters UNIFI™ Scientific Information System software version 1.7 (Waters, USA) for compounds identification.

**Specifications Table**

**Table:** 

Subject	Chemistry
Specific subject area	Phytochemicals, Natural product research, Spectrometry
Type of data	Tables, Figures
How data were acquired	Data on the phytochemical compounds in the aqueous extract of *S. polyanthum* (Wight) Walp. leaves were acquired using liquid chromatography quadrupole-time-of-flight mass spectrometry (Vion IMS LCQTOF MS 2016 model (Waters,USA)) using a reversed-phase column to separate and identify the semipolar to polar compounds.
Data format	Raw and Analyzed
Parameters for data collection	The data on the phytochemical compounds in *S. polyanthum* (Wight) Walp. leaves were acquired using Vion IMS LCQTOF MS 2016 (Waters,USA) with a reversed-phase column (ACQUITY UPLC HSS (2.1 × 100 mm × 1.8 µm) with the following operating conditions: operation mode (negative), analyzer mode (sensitivity), desolvation gas flow rate (600 L/h), cone gas (50 L/h), desolvation temperature (550 °C), source (electrospray ionization), source temperature (120 °C), capillary voltage (2.50 kV), MS mode (high definition), and scanning range (50–1000 m/z). The Auto MS/MS mode was used to confirm the fragment ions. The mass spectral features from samples raw data were matched with Traditional Medicine (en) and Waters Screening libraries in the Waters UNIFI™ Scientific Information System software version 1.7 (Waters, USA) for compounds identification.
Description of data collection	The freeze-dried powder of *S. polyanthum* (Wight) Walp. leaves aqueous extract was reconstituted in distilled water and then analyzed with Vion IMS LCQTOF MS 2016 model (Waters, USA) and then matched with Traditional Medicine (en) and Waters Screening libraries in the Waters UNIFI™ Scientific Information System software version 1.7 (Waters, USA) for compounds identification.
Data source location	*S. polyanthum* (Wight) Walp. leaves were collected from Sultan Haji Ahmad Shah Agricultural Park, Kuantan, Pahang, Malaysia (Latitude: 3.8470088044445445, Longitude: 103.30165631277539), and then extracted at the Natural Product Laboratory, Kulliyyah of Science, International Islamic University Malaysia, Kuantan, Pahang, Malaysia. LC-QTOF-MS/MS analysis and data processing were conducted at Central Laboratory, Universiti Malaysia Pahang, Kuantan, Pahang, Malaysia.
Data accessibility	The complete dataset is accessible at the Mendeley Repository: http://dx.doi.org/10.17632/8mt9npzyp8.1[4].

**Value of the Data**

• This dataset is essential to show the phytochemical constituents in *Syzygium polyanthum* (Wight) Walp. leaves, a variant collected from Kuantan, Pahang, a region in the East Coast of Peninsular Malaysia.
• This dataset provides valuable information to the ethnobotanist, plant chemist, taxonomist, and herbal medicinal plants researchers on this plant variant since there were reported chemical composition variations between the variants of the same plant species with different geographical locations that will affect the therapeutic potential of this plant [2,3].
• The dataset on the plant's chemical profile helps in fostering development of high-quality herbal medicinal products with standardized bioactive compound [5,6].

## 1 Data Description

**Fig. 1:**
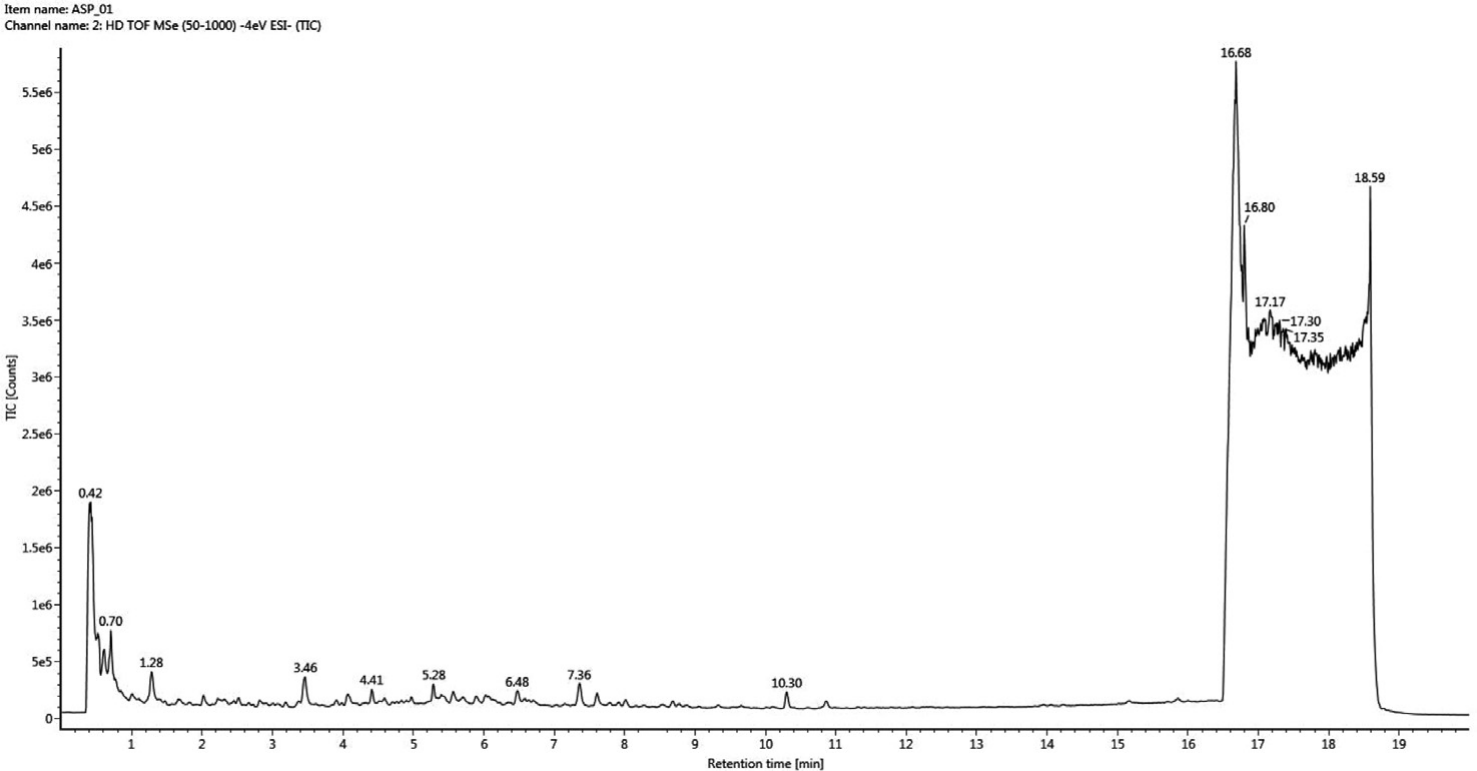
LCMS Chromatogram of *Syzygium polyanthum* (Wight) Walp. leaves aqueous extract.

**Fig. 2:**
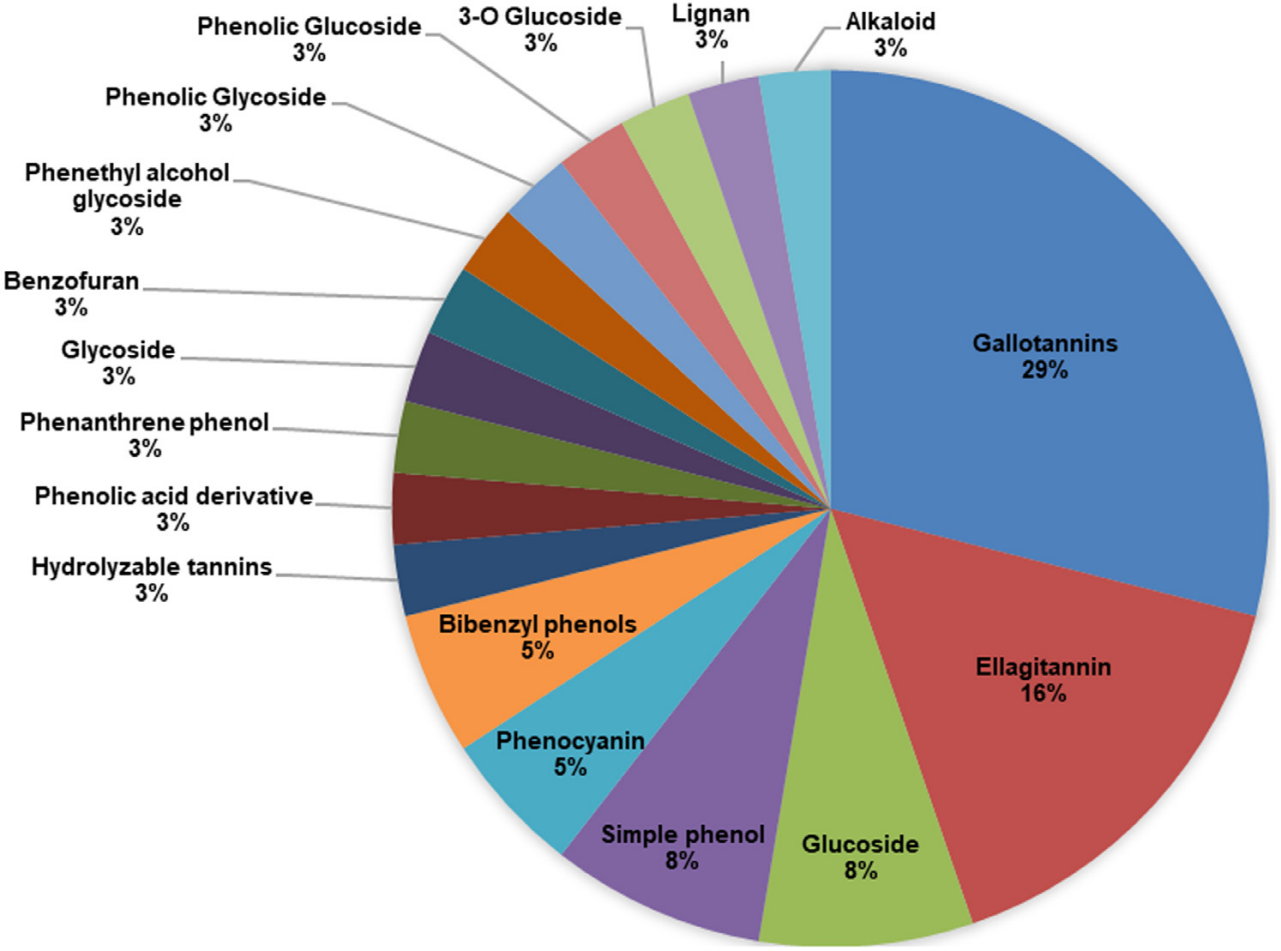
Percent distribution of phytochemical groups in *Syzygium polyanthum* (Wight) Walp. leaves aqueous extract.

Fig. 1 illustrates the total ion chromatogram of the sample. The LC-MS raw datasets were further matched with the Traditional Medicine (en) and Waters Screening libraries in the Waters UNIFI™ Scientific Information System software version 1.7 (Waters, USA) for compounds identification as tabulated in Table 1. The raw data of all identified compounds in the aqueous extract from the leaves of *S. polyanthum* (Wight) Walp. can be found in the Mendeley data repository at: http://dx.doi.org/10.17632/8mt9npzyp8.1 [4]. Supplement 1 shows the complete raw data report as generated by UNIFI. The report contains operating parameters, total ion current (TIC) plot, base peak intensities (BPI) plot, summary table of identified compounds which includes chemical formula, orserved neutral mass (Da), observed mass to charge ration (*m/z*), mass error (in mDa and ppm), observed retention time (min), responses, adducts, observed collision cross-section or CCS (Å²), total fragments found, and the spectral figures for each identified compound. Supplement 2 shows the simplified list of identified compounds in this plant extract. Fig. 2 shows percent distribution of phytochemical groups of identified compounds in this plant extract.

**Table 1:** Chemical compounds identified in *Syzygium polyanthum* (Wight) Walp. leaves aqueous extract using LC-QTOF-MS analysis.

No	Component name	Formula	Identifica-tion status	Observed neutral mass (Da)	Observed m/z	Mass error (mDa)	Mass error (ppm)	Observed RT (min)	Observed CCS (Å²)	Response	Adducts	Phytochemical Groups
1	2,6-Di-O-galloyl-β-D-glucose	C20H20O14	Identified	484.0824	483.0752	−2.9	−5.9	0.59	165	1110	−H	Gallotannin
2	1-Galloyl-glucose	C13H16O10	Identified	332.0749	331.0676	0.6	1.7	0.73	172.73	2695	−H	Gallotannin
3	1-Galloyl-glucose	C13H16O10	Identified	332.0746	331.0673	0.3	0.8	0.99	216.32	1060	−H	Gallotannin
4	1-Galloyl-glucose	C13H16O10	Identified	332.0746	331.0673	0.2	0.7	0.99	172.42	8288	−H	Gallotannin
5	2,3-(S)-Hexahydroxydiphenoyl-D-glucose	C20H18O14	Identified	482.0705	481.0632	0.8	1.7	1.01	200.74	2209	−H	Hydro-lyzable tannin
6	Gemin D	C27H22O18	Identified	634.0814	633.0741	0.8	1.2	1.12	222.57	2639	−H	Ellagitannin
7	1-Galloyl-glucose	C13H16O10	Identified	332.0748	331.0675	0.4	1.3	1.25	175.04	10088	−H	Gallotannin
8	Pyrogallic acid	C6H6O3	Identified	126.0318	125.0245	0.1	0.7	1.3	130.77	3260	−H	Phenolic Acid Derivative
9	Polygoaceto-phenoside	C14H18O10	Identified	346.0905	345.0832	0.5	1.3	1.42	173.81	2846	−H	Glucoside
10	1-Galloyl-glucose	C13H16O10	Identified	332.0747	331.0674	0.4	1.1	1.47	172.23	2168	−H	Gallotannin
11	Norbergenin	C13H14O9	Identified	314.064	359.0622	0.2	0.6	1.58	178.19	1424	+HCOO	Glycoside
12	1-Galloyl-glucose	C13H16O10	Identified	332.0744	331.0672	0.1	0.3	1.83	177	1366	−H	Gallotannin
13	5-Desgalloylstachyurin	C34H24O22	Identified	784.0755	783.0682	−0.5	−0.6	2.02	254.89	4623	−H	Ellagitannin
14	1-Galloyl-glucose	C13H16O10	Identified	332.0747	331.0674	0.3	1	2.24	168.93	1366	−H	Gallotannin
15	5-Desgalloylstachyurin	C34H24O22	Identified	784.0759	783.0686	−0.1	−0.1	2.45	256.87	1555	−H	Ellagitannin
16	2,6-Di-O-galloyl-β-D-glucose	C20H20O14	Identified	484.0854	483.0782	0.1	0.3	3.19	196.43	3851	−H	Gallotannin
17	2,4,5-Trihydeoxybenzaldehyde	C7H6O4	Identified	154.0265	153.0192	−0.1	−0.9	3.46	171.96	1230	−H	Simple phenol
18	5-Desgalloylstachyurin	C34H24O22	Identified	784.0753	783.068	−0.7	−0.8	3.59	257.26	1216	−H	Ellagitannin
19	Haematoxylin	C16H14O6	Identified	302.079	347.0772	0	−0.1	3.87	179.52	1141	+HCOO	Phenocyanin
20	2,6-Di-O-galloyl-β-D-glucose	C20H20O14	Identified	484.0855	483.0782	0.2	0.4	4.2	195.97	4799	−H	Gallotannin
21	2,6-Di-O-galloyl-β-D-glucose	C20H20O14	Identified	484.0855	483.0782	0.2	0.3	4.29	193.82	3329	−H	Gallotannin
22	Mulberrofuran C	C34H28O9	Identified	580.1742	579.1669	0.8	1.4	4.42	223.24	1802	−H	Benzofuran
23	1-O-Galloylpedun-culagin	C41H28O26	Identified	936.0862	935.0789	−0.7	−0.7	4.83	295.29	8312	−H	Ellagitannin
24	Darendoside A	C19H28O11	Identified	432.1631	431.1558	0	−0.1	5.14	211.06	1402	−H	Phenethyl alcohol glycoside
25	Aspidinol	C12H16O4	Identified	224.1046	269.1028	−0.3	−1	5.44	203.8	1138	+HCOO	Simple phenol
26	Feroxin A	C17H24O8	Identified	356.1472	401.1454	0.1	0.1	5.44	202.21	15156	+HCOO	3-O Glucoside
27	Dendrocandin D	C17H20O5	Identified	304.1289	349.1271	−2.2	−6.3	5.71	182.04	2016	+HCOO	Bibenzyl phenols
28	Brazilein	C16H12O5	Identified	284.069	329.0672	0.6	1.7	5.91	169.26	1333	+HCOO	Phenocyanin
29	2,3-(S)-Hexahydroxydiphenoyl-D-glucose	C20H18O14	Identified	482.0702	527.0684	0.5	1	6.49	210.74	1336	+HCOO	Ellagitannin
30	Isotachioside	C13H18O8	Identified	302.1	301.0928	−0.1	−0.5	7.91	195.15	1637	−H	Phenolic Glycoside
31	Tachioside	C13H18O8	Identified	302.1	301.0928	−0.1	−0.5	7.91	195.15	1637	−H	Phenolic Glucoside
32	Thannilignan	C19H22O5	Identified	330.1464	329.1391	−0.3	−1	8.03	186.08	1238	−H	Lignan
33	2,3,5,4′-Tetrahydroxystilbene-2-O-(6′'-O-α-D-glucopyranosyl)-β-D-glucopyranoside	C26H32O14	Identified	568.1792	567.1719	0	−0.1	11.59	224.46	1544	−H	Glucoside
34	Aspidinol	C12H16O4	Identified	224.1048	223.0975	−0.1	−0.4	12.34	200.45	1323	−H	Simple phenol
35	2′-Hydroxy-3′,4′-dimethoxy-isoflavan-7-O-β-D-glucoside	C23H28O10	Identified	464.164	509.1622	−4.2	−8.3	16.58	221.66	1885	+HCOO	Glucoside
36	Nisoldipine	C20H24N2O6	Identified	388.1645	387.1572	1	2.7	16.99	205.15	1353	−H	Alkaloid
37	Yakuchinone A	C20H24O3	Identified	312.1757	311.1684	3.1	10	18.56	189.48	6985	−H	Bibenzyle phenol
38	2,7-Dihydroxy-4-methoxyphenanthrene-2-O-glucoside	C21H22O8	Identified	402.1354	447.1336	3.9	8.7	18.64	214.4	1064	+HCOO	Phenanthrene phenol

## 2 Experimental Design, Materials and Methods

### 2.1 Plant collection, authentication, and preparation of plant material

*S. polyanthum* (Wight) Walp. leaves were collected from Sultan Haji Ahmad Shah Agricultural Park, Kuantan, Pahang, Malaysia in May, 2019. The dried leaves samples were deposited into Kulliyyah of Pharmacy Herbarium, International Islamic University Malaysia, with the voucher number of PIIUM-0282-1 and were identified as *Syzygium polyanthum* (Wight) Walp. For the plant's extraction, the leaves were allowed to dry for three weeks at room temperature. The dried leaves were then grinded into powder, macerated in distilled water at 80 °C for 30 min in a bath-sonicator (WiseClean, Switzerland) with the wavelength range of 40–80 λ. The macerated leaves were then filtered using Whatmann filter paper No. 1, and then the filtrate was freeze-dried for a week in a freeze-dryer (CHRIST Model Beta 1-8 LO, Germany) before LC-MS analysis.

### 2.2 Phytochemical profiling of the aqueous extract of S. polyanthum (Wight) Walp

Phytochemical profiling of the aqueous extract of *S. polyanthum* (Wight) Walp. leaves was conducted using an LC-MS instrument, VION Ion Mobility QTOF-MS (Waters, USA) based on the method adopted from our previous study [7]. The crude extract (1 mg) was firstly dissolved in 1 mL distilled water before being filtered using a filter membrane (25 mm diameter, 0.45 µm pore size). 10 µL of sample was then injected into a reversed-phase ACQUITY UPLC HSS (2.1 × 100 mm x 1.8 µm) column system with a binary pump of two different solvents comprising Solvent A and B with the compositions as shown in Table 2. The operating parameters used in the analysis are presented in Table 3.

**Table 2:** Gradient flow profiles of the mobile phase.

Time (min)	Flow (µL/min)	Solvent A (Water with 0.1% Formic acid)	Solvent B (Acetonitrile with 0.1% Formic acid)
0	0.5	99%	1%
0.5	0.5	99%	1%
16	0.5	65%	35%
18	0.5	0%	100%
20	0.5	99%	1%

**Table 3:** Operating parameters of Vion IMS QToF analyzer.

Operating parameters	Values
Operation mode (Polarity)	Negative (–ve)
Analyzer Mode	Sensitivity
Source type	Electrospray ionization (ESI)
Source temperature	120 °C
Desolvation temperature	550 °C
Desolvation gas flow rate	600 L/h
Cone gas	50 L/h
Capillary voltage	2.50 kV
Scan time	0.200 s
MS mode	High-definition MS
Scanning range	50–1000 m/z
Column type	ACQUITY UPLC-BEH (Waters, USA)
Dimension	C18, 2.1 × 100 mm

### 2.3 Data acquisition, processing, and reporting

In this study, the Waters® UNIFI [1.7] Scientific Information System with embedded Traditional Medicine library as well as Waters® Screening library databases were utilized for data acquisition, data mining, library searching and report generation. This system utilized the raw data including individual chromatographic peaks, retention time, mass-to-charge ration (*m/z*), and spectral resolution to match with the library and then generated the summary of identified compounds alongwith data for its chemical formula, orserved neutral mass (Da), observed mass to charge ration (*m/z*), mass error (in mDa and ppm), observed retention time (min), responses, adducts, observed collision cross-section or CCS (Å²), total fragments found, and the spectral figures for each identified compound. Only identified components with mass error of less than 5 mDa were included in the list http://dx.doi.org/10.17632/8mt9npzyp8.1.

## Data Availability

Phytochemical Compounds in Syzygium polyanthum (Wight) Walp. Leaves Collected from Kuantan, Pahang, Malaysia: LCMS dataset (Original data) (Mendeley Data).

## Ethics Statement

This research work does not require ethical approval.

## CRediT authorship contribution statement

**T.A. Faiz T. Anuar:** Writing – original draft, Formal analysis. **Azlini Ismail:** Conceptualization, Methodology, Writing – original draft. **Izzat Fahimuddin Mohamed Suffian:** Supervision, Validation. **Azzmer Azzar Abdul Hamid:** Writing – review & editing. **Mohd Hafiz Arzmi:** Writing – review & editing. **Muhammad Nor Omar:** Supervision, Validation, Writing – review & editing.

## Declaration of Competing Interest

The authors declare that they have no known competing financial interests or personal relationships which have or could be perceived to have influenced the work reported in this article.
